# Application of MCMC-Based Bayesian Modeling for Genetic Evolutionary and Dynamic Change Analysis of Zika Virus

**DOI:** 10.3389/fgene.2019.01319

**Published:** 2020-01-10

**Authors:** Tong Shao, Jiahui Pan, Shiwei Zhang, Zhuoyuan Xin, Guoqing Wang

**Affiliations:** ^1^Department of Pathogenobiology, The Key Laboratory of Zoonosis, Chinese Ministry of Education, College of Basic Medical Science, Jilin University, Changchun, China; ^2^The Key Laboratory for Bionics Engineering, Ministry of Education, China, Jilin University, Changchun, China

**Keywords:** zika virus, NS5, evolution, dynamic changes, Bayesian method

## Abstract

Zika virus was first discovered in 1947. For a long time afterward, no large-scale outbreaks occurred. However, more recently, in 2007 and 2016, there were two episodes of ZIKV outbreak that have produced serious public health problems. By analyzing the evolution of the viral genome, we can understand the potential for its outbreak. In this study, we constructed a maximum clade credibility (MCC) tree for the ZIKV non-structural protein 5 (NS5) gene using the Bayesian method. A total of 108 whole-NS5 sequences were retrieved from the GeneBank. We carried out an analysis of potential glycosylation and phosphorylation sites of the ZIKV virus NS5 gene and dynamic analysis of the evolutionary characteristics of the gene. Phylogenetic analysis revealed the presence of two sequence lineages: African and Asian. The sequence of the strains obtained from GeneBank has high homology of 85% to 100%. There are 35 potential phosphorylation sites and glycosylation sites in the ZIKV-NS5 sequences. This article analyzes the possible causes of ZIKV virus outbreaks from the perspective of genetic evolution and analyzes the dynamic trends of virus outbreaks to provide a theoretical basis for predicting the outbreak of the virus.

## Introduction

In 1947, Zika virus (ZIKV) was first isolated from a monkey in Zika forest, Uganda. (Dick et al., 1952). ZIKV is a member of the virus family *Flaviviridae* and genus *Flavivirus* and is a mosquito-transmitted virus. The virus particles are spherical, with diameters of about 40 ~ 70 nm. Zika virus is a type of single-stranded, positive-sense RNA virus. The whole-genome length is about 10.8 kb, and its single ORF encodes three structural proteins and seven non-structural proteins (NS1, NS2A, NS2A, NS4A, NS4A, NS4B, and NS5) (Kuno and Chang, 2007). The nonstructural protein 5 (NS5) is necessary for genomic replication of zika virus. The N-terminal of NS5 contains methyltransferase (MT), followed by the RNA-dependent RNA polymerase (RdRp). The methyltransferase domain at the N-terminal stabilizes the viral RNA genome through 5' capping, while the RdRp domain at the end of C-terminal is very important for the RNA replication of the virus (Decroly et al., 2011; Lu and Gong, 2013; Zhao et al., 2015).

The main means of transmission of ZIKV is through *Aedes* mosquito bites, perinatal transmission, sexual contact, and blood transfusion (Besnard et al., 2014; Musso et al., 2014; Franchini and Velati, 2016). Since the first discovery of Zika virus in 1947, it has gradually spread to become a large-scale problem in the world. The first strain isolated from Asia was named “P6–740” and was isolated from Aededon in Malaysia in 1966 (Haddow et al., 2012). Molecular biology and bioinformatics analysis showed that there are two subtypes of ZIKV, the African and Asian lineages. However, from 1966 to 2007, confirmed cases were scarce, and there was no associated sequence data regarding the Asian linkage. That was until 2007, when 49 cases of ZIKV infection were confirmed in Yip Island and became the first large-scale human infection event in history (Duffy et al., 2009). Now, more than 30 countries have reported ZIKV infections, and these infections have led to multiple imported cases. The ZIKV epidemic has become an important public issue of concern to the whole world (Gong et al., 2016). Base variation, including base recombination, conversion and deletion, will affect the codon usage pattern of the virus, and changes in the codon usage pattern will affect the encoded protein. It is reported that there are potential mutation sites associated with microcephaly (Wang et al., 2017). Studies have shown that envelope protein and NS1 protein of zika virus are predicted to have glycosylation modification sites (Lanciotti et al., 2008; Haddow et al., 2012; Faye et al., 2014). Recently, it has been suggested that correlation between the polymorphism of glycosylation sites and vectors has caused the evolution of Zika virus (Faye et al., 2014). The prediction of viral mutation sites and glycosylation sites is of great significance for understanding the evolution of the virus and the spread of the disease.

Bayesian Inference (BI) is based on using the evolutionary model of sequence evolution to reconstruct the statistical method of the system tree. The resulting tree not only reflects the best estimate of the phylogenetic relationship but also provides the exact support for the branch (Battaglia et al., 2016). Because of the important function of the NS5 gene and the previous construction of an evolutionary tree using the NS5 gene (Gong et al., 2016; Shen et al., 2016), this article uses the Bayesian method to analyze the evolution of the Zika virus NS5 protein, with simultaneous analysis of possible mutation sites. The research result provides a significant theoretical guide to the prevention and treatment of the disease.

## Materials and Methods

### Sequence Collection

The total of 108 NS5 gene sequences that had been added to GenBank before October 2017 were downloaded for Bayesian analysis. These gene sequences are the complete NS5 sequences. The detailed sequence information is listed in Additional **Table S1**.

### Sequence Analysis and Comparisons of NS5

The nucleotide sequences of Zika virus NS5 were analyzed using DNASTAR Lasergen 7.0 software to compare their homology.

### Analysis of Potential Protein Modification Sites

We used the NetOGlyc 4.0 Server (http://www.cbs.dtu.dk/services/NetOGlyc/) (Steentoft et al., 2013) to estimate the O-glycosylation status of these NS5 sequences (Boon et al., 2016). GlycoMine was used to predict the C-linked and N-linked glycosylation (http://glycomine.erc.monash.edu/Lab/GlycoMine/).

### Analysis of the Obtained Viral Genome Data by the Bayesian Method

The Bayesian analysis method was used to study the present evolution rate and evolution model of the epidemic ZIKV strain. The complete NS5 sequence alignment of the ZIKV was disposed carefully with the Clustal W program in MEGA. The RDP3 recombination package was used to detect the recombination of all the sequences. The saturation monitoring was also tested by screening sequences with DAMBE software. If ISS < ISS.c, it means that the sequence substitution is not saturated and meets the requirements for building a phylogenetic tree using Bayesian methods. Finally, the best evolution model was selected with jModelTest software. BEAST v1.8.0 was employed under the GTR+I+G model of nucleotide substitutions and with the Relaxed clock:Uncorrelated Log-normal setting to perform 80 million MCMC runs to construct a maximum clade credibility tree (effective sampling size >200). The analysis was sampled at every 8000 states. Posterior probabilities were calculated with a burn-in of 8 million states. The analysis of the sampling data was output by Tracer v1.6, and the Tree Annotator program was employed to output the results of the MCC tree model. FigTree program was then used to plot the MCC molecular evolutionary tree.

## Results

### Homologous Comparison of Zika Virus Sequences

**Figure 1:**
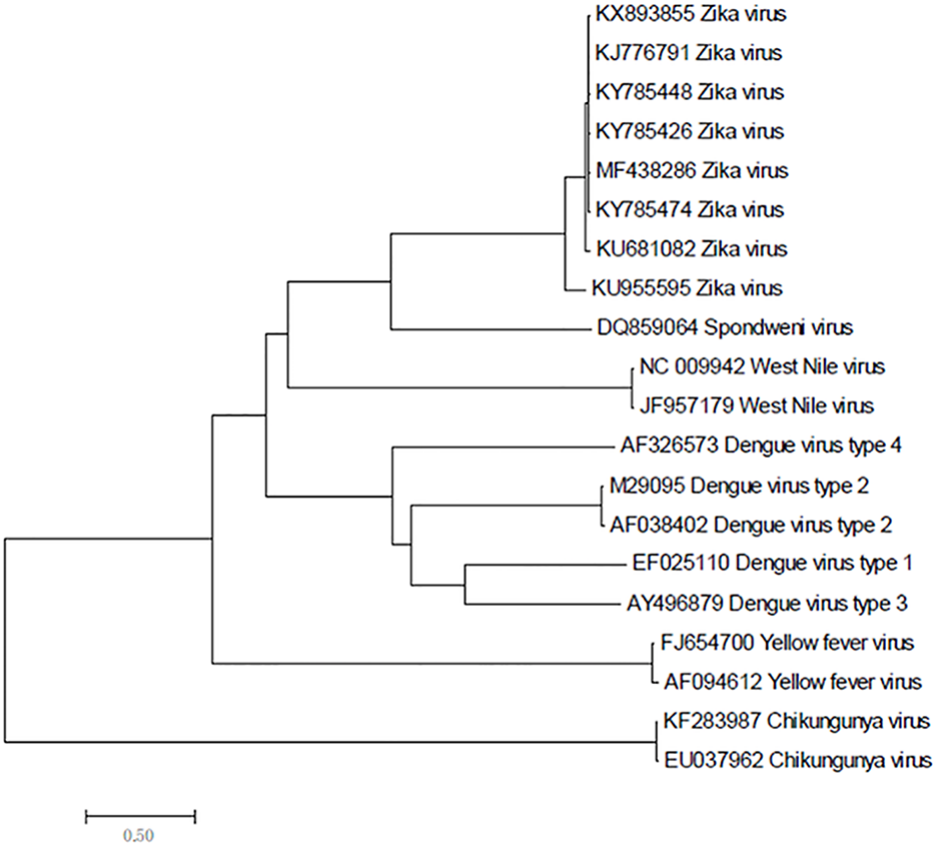
Phylogenetic analysis of Zika virus, Dengue virus, Spondweni virus, West Nile virus, Yellow fever virus and Chikungunya virus based on NS5 gene.

**Figure 2:**
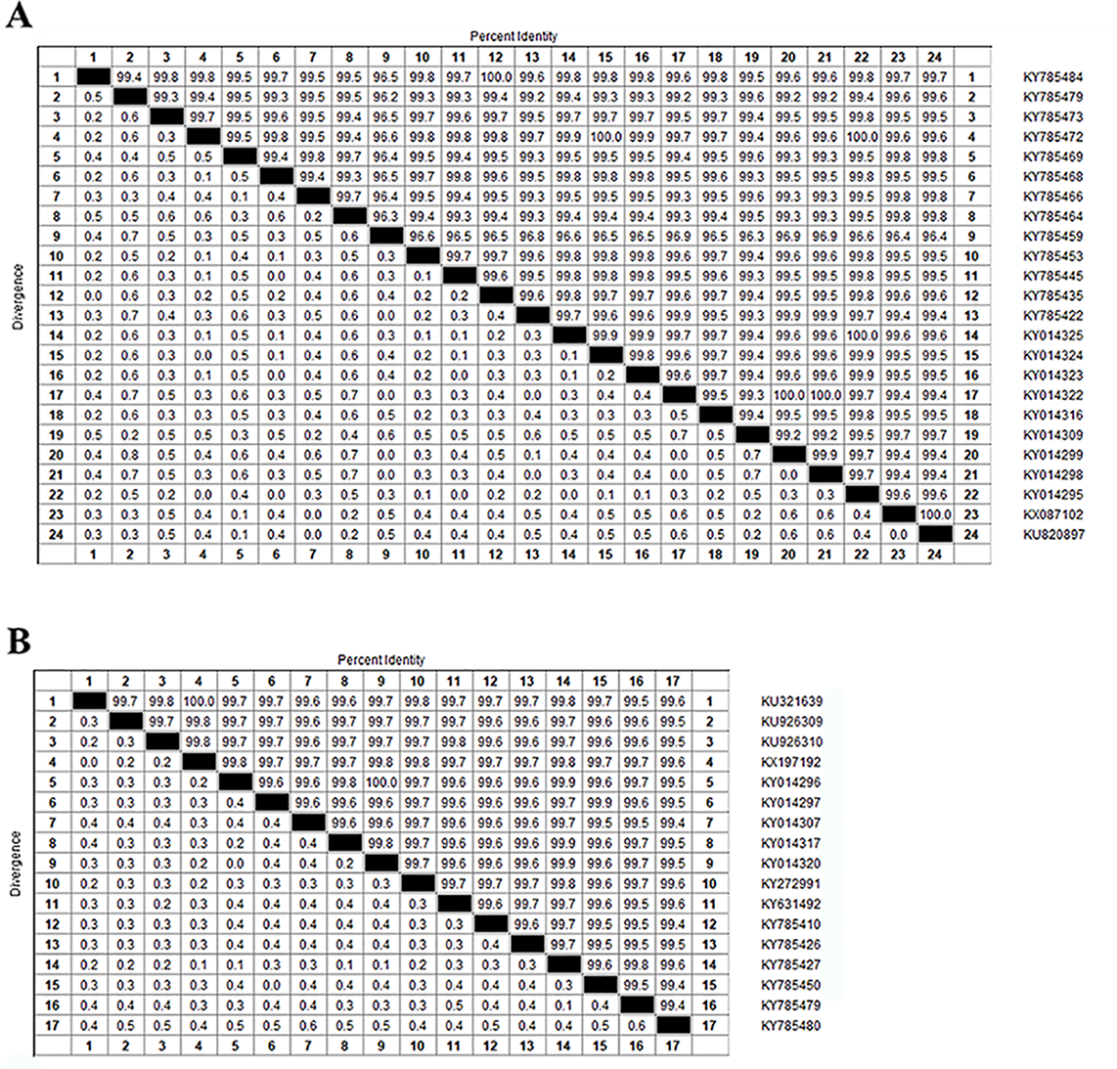
Homologous comparison. **(A)** Homology alignment analysis of 24 Zika strains from the United States using the DNASTAR software package. **(B)** Homology alignment analysis of 17 Zika strains from Brazil using the DNASTAR software package.

Zika virus is a member of the family *Flaviviridae* and genus Flavivirus and is a mosquito-transmitted virus. In the phylogenetic tree, it is close to Dengue virus, Japanese encephalitis virus, and West Nile virus;, the closest virus is Spondweni (**Figure 1**). The Zika virus strains used in this study were 108 strains collected from 20 districts. The results showed that the nucleotide homology of the 108 strains of Zika virus was between 85.4% and 100% and that some of them were 100% homologous. Twenty-four strains from the United States (**Figure 2A**) and seventeen strains from Brazil are compared respectively (**Figures 2B**). We can see the homogeneity of the twenty-four strains of the NS5 gene from the United States is 96.3%~100%, and there are many sequences of the strains of the NS5 amino acid that have a homology of 100%. The same result was found in the Brazil strains. The nucleotide homology of the Brazil strains is 99.4%~100%. This result shows that the mutation rate in the 108 strains is low and that the NS5 gene is relatively conservative.

### Prediction of Glycosylation Sites

**Figure 3:**
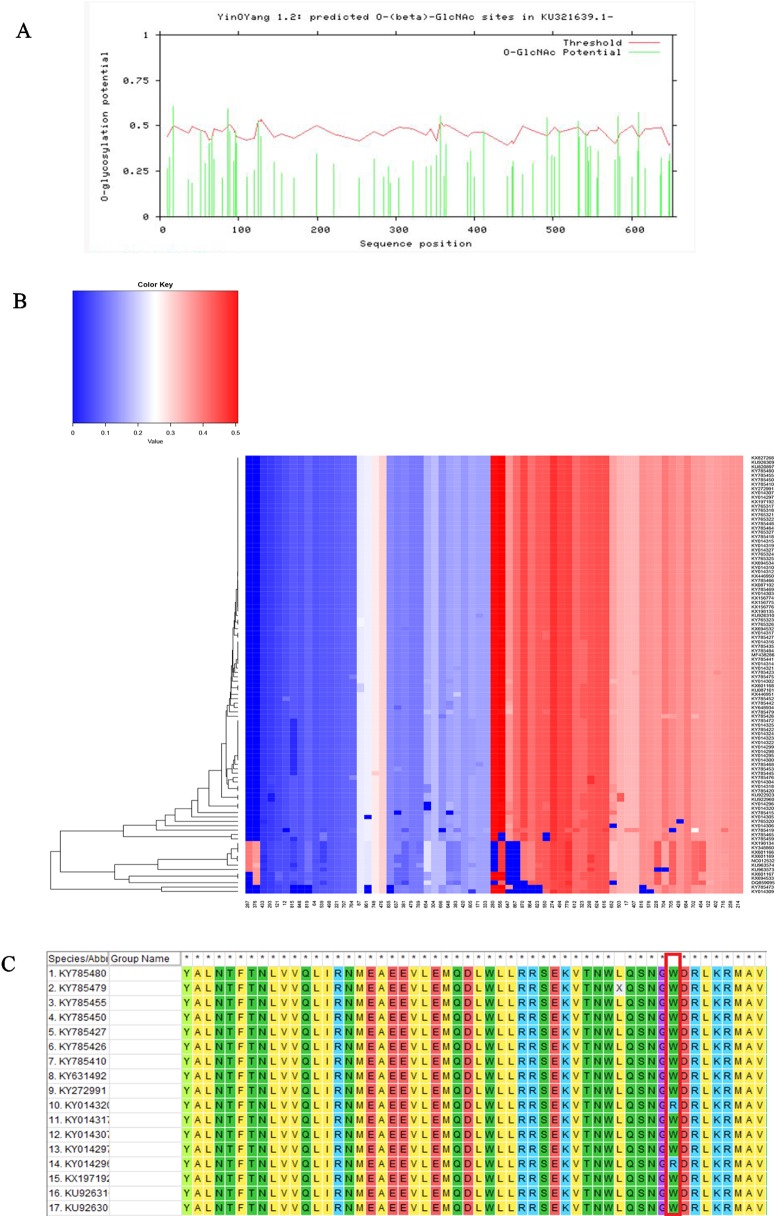
Functional site prediction. **(A)** Prediction of O-glycosylation site of Zika virus from Brazil using the NetOGlyc website. **(B)** Heat map of predicted N-linked and C-linked glycosylation sites. The ordinate of the heat map represents the sequence of different strains; the abscissa represents all amino acid sites of the NS5 gene. Glycosylation predictions are performed on 100 sequences. The darker the red color, the higher the score. **(C)** Sequence alignment result.

We performed three types of glycosylation site prediction for NS5 sequences of 100 strains. We used GlycoMine to predict C-linked and N-linked glycosylation sites and NetOGlyc to predict O-glycosylation. It can be seen from the results that there are 10 sites that are potentially modified by O-glycosylation (**Figure 3**), and the number of sites of N-linked and C-linked glycosylation that may occur in different strains is not much different (**Figure 3**). For example, comparing one strain of KY014296 from Brazil with other strains from Brazil that lack a C-linked site 654 (**Figure 3**), we can see in the sequence alignment that the amino acid of the strain at this position is arginine, whereas the amino acid of the other strains at this position is tryptophan.

### Recombinant Analysis of Virus Strains

**Figure 4:**
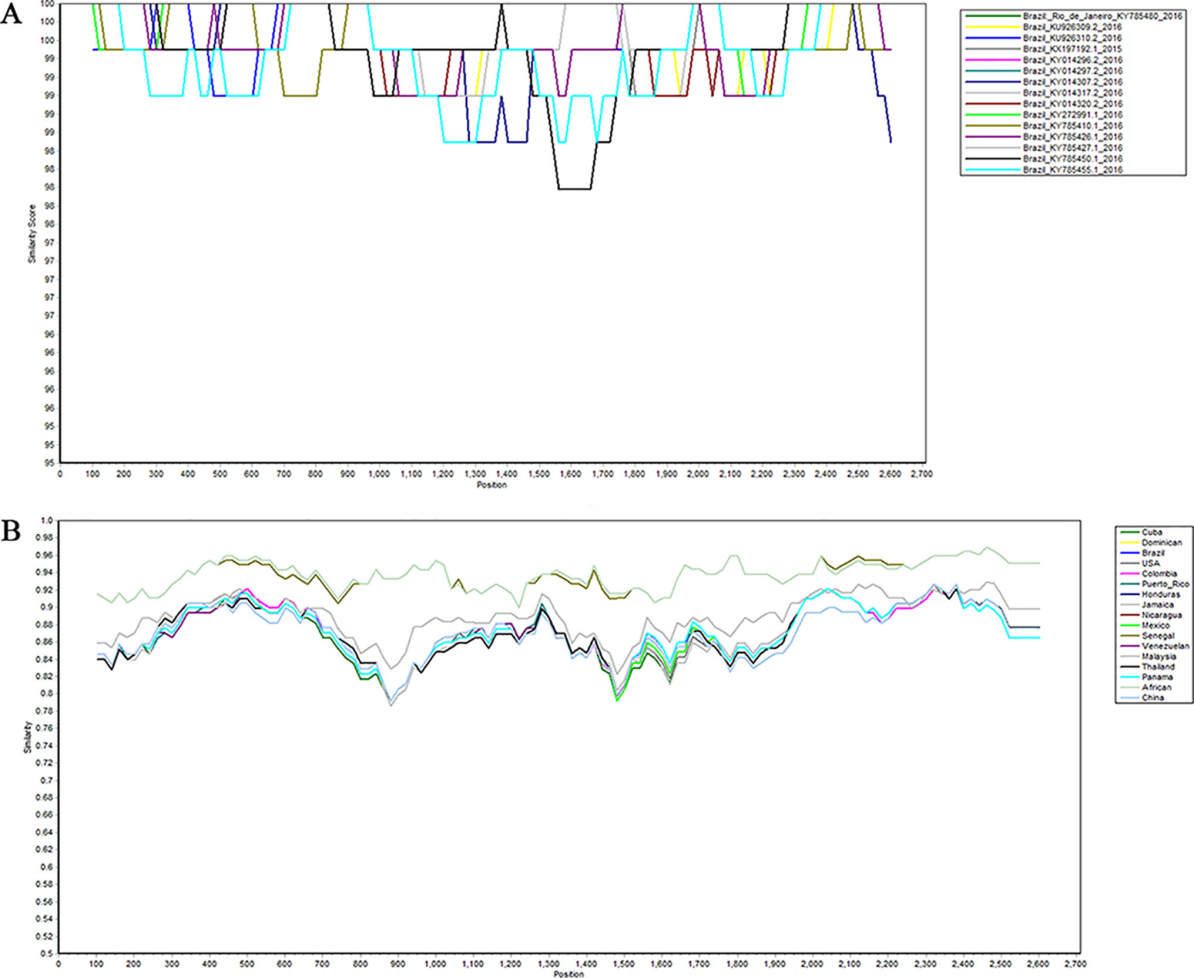
Recombination analysis of Zika virus. Plots of the similarity (generated by SimPlot) of a set of reference sequences. **(A)** Sequences of Zika virus from Brazil. **(B)** Comparison after grouping the Zika virus into different regions.

In order to identify whether recombination occurs between different strains in the same region, we used SimPlot to analyze the sequences of different strains in the same region. As shown in **Figure 4**, there is no recombination in the strains of Brazil. The sequences of all of the NS5 genes were then grouped by region, with the strains from the same region grouped together. SimPlot was then used to verify the occurrence of recombination events further. From **Figure 4**, we can see that there is no recombination event in any of the sequences. The above results indicate that no recombination occurred in the selected strain sequences.

### Evolutionary Tree Construction Based on the Bayesian-Markov Chain Method

**Figure 5:**
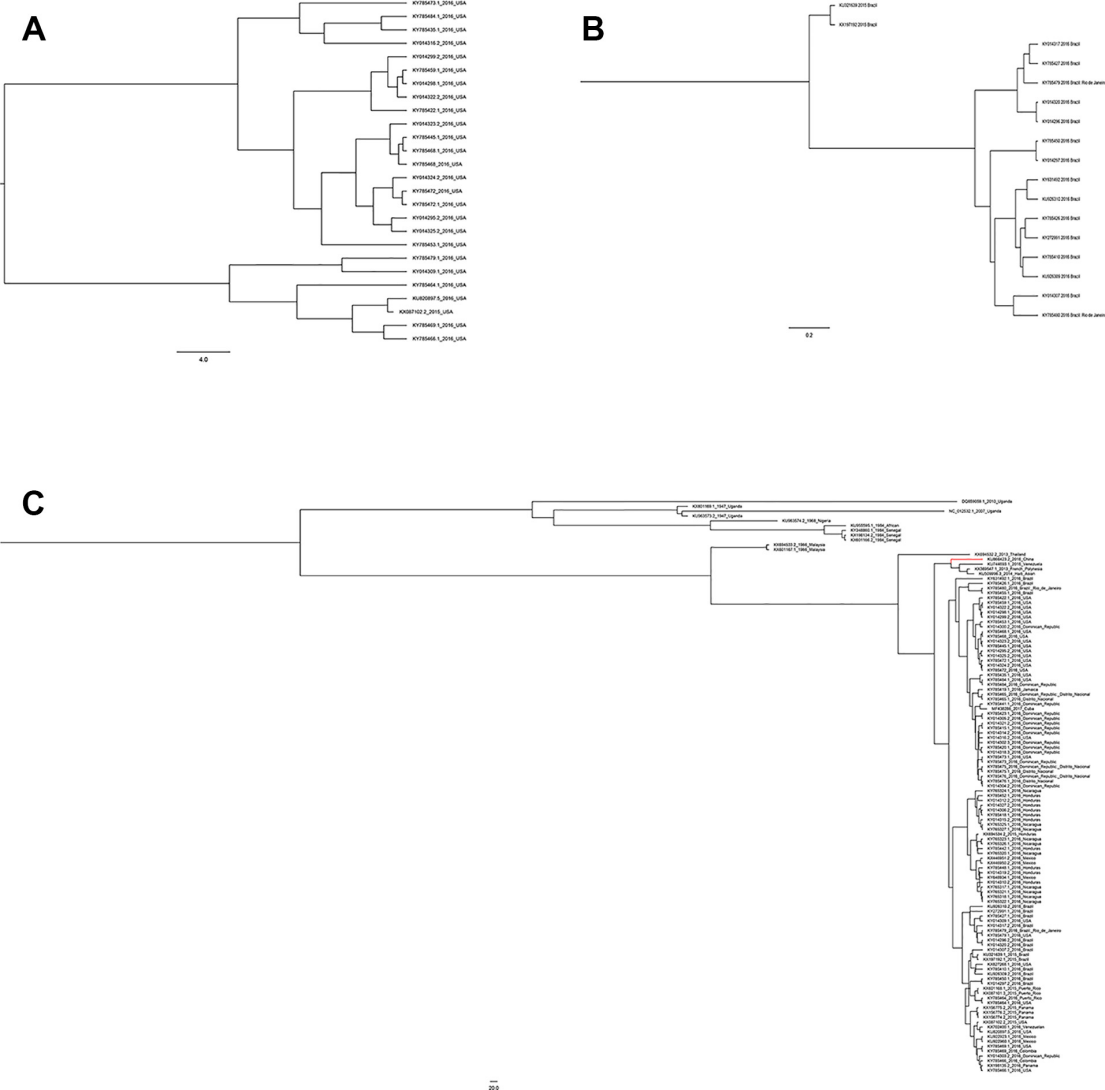
Phylogenetic analysis of Zika virus. **(A)** Evolutionary development of 24 strains of Zika virus NS5 genes from the United States. **(B)** Evolutionary development of 17 strains of Zika virus NS5 genes from Brazil. **(C)** Evolutionary development of 108 strains of Zika virus NS5 genes.

A total of 108 complete NS5 gene sequences were used in the phylogenetic analysis. Samples were collected from 20 regions. Although these strains were from 20 different regions, they were eventually divided into two groups, the African lineage and the Asian lineage. After 2015, the isolated strains were very close to each other and the new outbreak strains selected in this study are all Asian-type. Indicating that during this time, the NS5 gene sequence of ZIKV was conservative. There is no extra genotype from after the outbreak of the ZIKV epidemics in 2015 and 2016. The recent outbreak was predominantly in Asia, and the contemporary epidemics are dominantly evolved from Asian strains. Neither the American strains nor the Brazilian strains have a very specific genotype (**Figure 5C**). Moreover, among these strains, there is no clear dividing line between the strains of each country. As can be seen from **Figure 5**, strains from the United States, Brazil, and the Dominican Republic are cross-distributed in the phylogenetic tree, with the closest ancestor being a tree root. Strains from Honduras, Nicaragua, and Mexico are closely spaced, and these are the strains most distant from the Africa linkage. From a temporal perspective, the kinship strains we collected at different times from the same area were the most recent. This shows that the strains in each region are from local ancestors, and there is no cross-infection with other regions.

**Figure 6:**
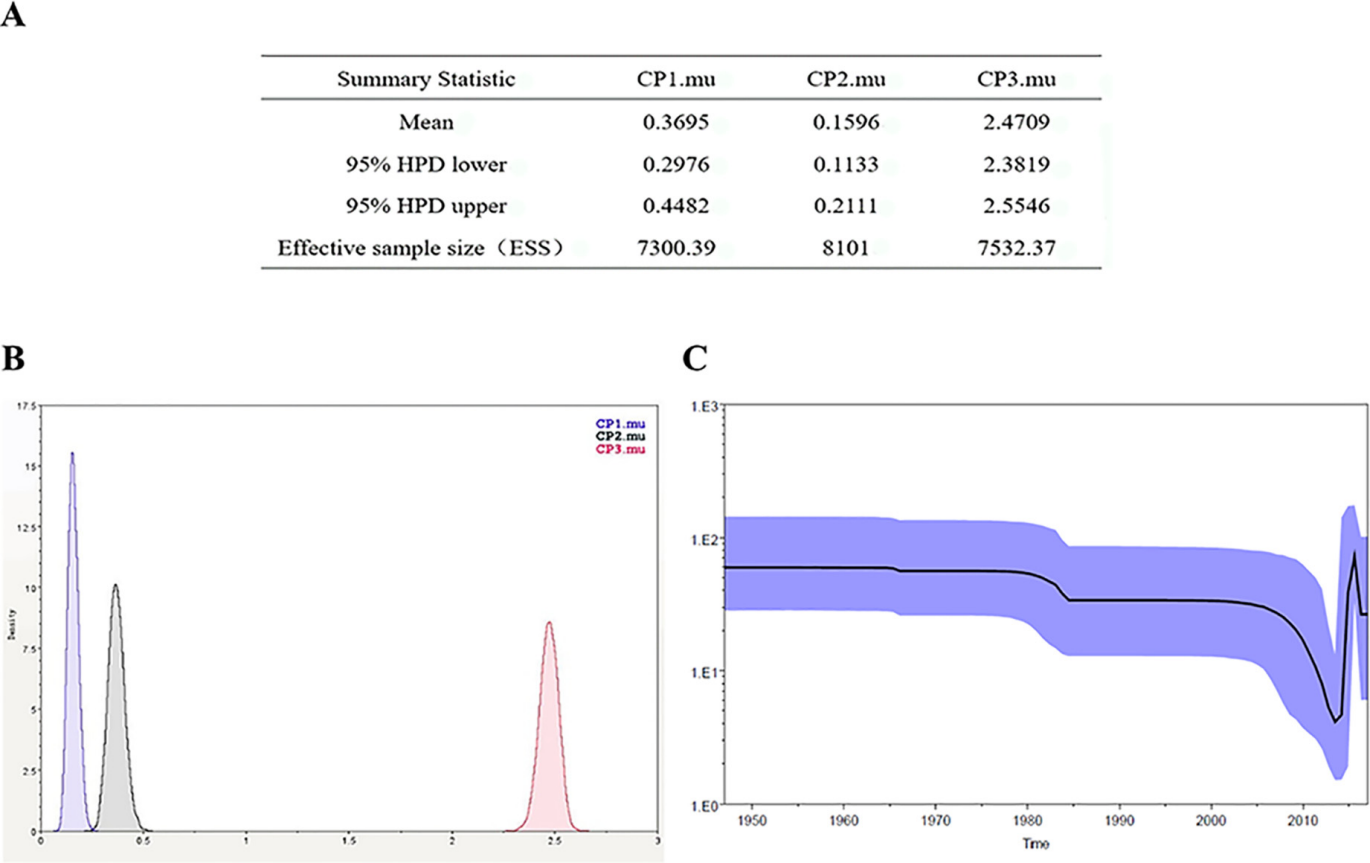
Zika virus NS5 codon mutation rate and skyline plot **(A**, **B)**. The codon mutation rate of the Zika virus NS5 gene was estimated by the Bayes-Markov chain method. The codon mutation rate is the result of a BEAST run using Trace analysis. **(C)** Dynamic study of Zika virus NS5 gene genetic diversity by Bayesian skyline plot. The thick solid line is the median estimate, and the dotted line shows the 95% confidence interval. The abscissa is time, and the ordinate is the effective population size. The curve shows that the Zika virus NS5 gene has been in a stable state and the population gradually began to grow in 2015.

The Bayesian-Markov chain method was used to determine the codon mutation rate of the Zika NS5 gene, and the BEAST results were analyzed by Trace. The results showed that the codon mutation rates of the amino acids encoded of ZIKV NS5 were different, and, respectively, the mutation rates of the three codons were 0.3695, 0.1596, and 2.4709 (**Figures 6A**). Thus, the mutation rate of the third codon was the highest. Since the mutation rate of the third codon is the highest and the codon has degeneracy, some mutations do not change the amino acids of the encoded protein, which makes the homology between the Zika virus strains very high. These values indicate that during this period, there was a base mutation of the NS5 gene, and this may be associated with the recent outbreak of the Zika virus. The geographical distribution of Zika viruses is steadily growing. As can be seen from the skyline plot (**Figure 6**), the effective size of the Zika virus has decreased since its discovery, but it also increased somewhat in 2015, coinciding with the Zika virus outbreak.

## Discussion

The Zika virus, which was discovered in 1947, returned 70 years after its discovery, unexpectedly appearing in the Pacific Islands and Latin America. Pathogenic changes, including microcephaly and Guillain-Barre syndrome, have caused widespread concern. One possible reason for this is the objective environmental conditions of an increased global population and an increased mosquito vector population (Pettersson et al., 2016; Shi et al., 2018). Another possibility is that amino acid substitution occurs that affects the rate of transmission and the pathogenicity of the virus. The effect of amino acid substitution on pathogenicity has been reported previously. For example, it was found that there was a substitution from S to N at position 139 of the prM protein before the French Polynesian outbreak of 2013 and that the subsequent strains in the Americas were all 139N. In vitro experiments showed that amino acid substitution enhanced infectivity and induced more severe microcephaly. Interaction between the virus and the host can lead to different infection outcomes (Yuan et al., 2017). Yang Liu et al. showed that spontaneous mutations on NS1 proteins increase their own antigenemia (Liu et al., 2017). Hongjie Xia et al. believe that mutations in Zika NS1 protein increase the body's ability to evade the immune response and increase the possibility of infection and epidemic (Xia et al., 2018). The replacement of one amino acid site has the potential to improve pathogenicity and transmission efficiency, which may explain why Zika virus has re-emerged after so many years. This is of great significance to study this mutation.

Compared with other gene fragments, NS5 and envelope gene fragments still had higher variability, although the non-structural proteins NS3 and NS5 were relatively conserved compared with other gene fragments according to homologous modeling analysis (Koh, 2014; Mazeaud et al., 2018), which in turn affects the genetic stability of the protein, making it easier for the virus to invade the human body (Yuan et al., 2015). We can observe an obvious cluster of NS5 genes consisting of only Chinese strains (**Figure 4** red), and the genetic distance between Chinese strains and French Polynesian strain is small. In 2013, a study showed that this strain from China and the Latin American strains have a common ancestor. (Faria et al., 2016). This suggests that this Chinese lineage may have evolved from an ancestor that erupted in the Pacific islands in 2013. Asian strains form an independent cluster, and the recent outbreaks of the Zika virus are of Asian lineage, indicating that Asian strains are more diversified than African Zika virus strains. There is a certain degree of mutation in the NS5 genes of Zika virus strains collected from Brazil and the United States. However, these mutations did not alter the glycosylation and phosphorylation sites of the NS5-encoded protein, suggesting that though there are mutations in the NS5 gene, these mutations did not impair the stability of the virus, and the protein structure, which plays an important role in the protein structure, remained stable. From **Figure 5**, we can see that the mutation rate of the third codon is the highest. Because of the degeneracy of the codon, mutations that occur on the third codon may not cause amino acid changes, which could explain why the various glycosylation and phosphorylation sites did not change after mutation. Glycosylation sites in the Zika virus genome display polymorphisms and may have adaptive value in evolutionary processes (Singh et al., 2016). It has been reported that Zika virus has a loss of glycosylation sites (Hanna et al., 2005; Lee et al., 2010). Mutations at amino acid sites play an important role in the pathogenicity of Zika virus, so analysis of the virus evolution is critical to better understand the pathogenesis of viral infection and the variability of its clinical phenotype.

The data we selected included the NS5 protein sequence of the Zika virus that broke out in 2016 and previously. The relatively stable NS5 gene nucleotide sequence will provide a great opportunity to develop a vaccine for this disease. We predicted the dynamic phylogenetic trends, which indicate the outbreak trends of ZIKV and provide theoretical foundations for clinical prevention. The potential glycosylation and phosphorylation sites of the NS5 gene were predicted and discussed in conjunction with existing functional assays.

## Data Availability Statement

Publicly available datasets were analyzed in this study. This data can be found here: https://www.ncbi.nlm.nih.gov/nuccore/?term = DQ859059, KU321639, KU509998, KU744693, KU820897, KU866423, KU922923, KU922960, KU926309, KU926310, KU955595, KU963573, KU963574, KX087101, KX087102, KX156774, KX156775, KX156776, KX197192, KX198134, KX198135, KX369547, KX446950, KX446951, KX601166, KX601167, KX601168, KX601169, KX694532, KX694533, KX694534, KX702400, KX827268, KY014295, KY014296, KY014297, KY014298, KY014299, KY014300, KY014302, KY014303, KY014304, KY014305, KY014306, KY014307, KY014309, KY014310, KY014312, KY014314, KY014315, KY014316, KY014317, KY014318, KY014319, KY014320, KY014321, KY014322, KY014323, KY014324, KY014325, KY014327, KY272991, KY348860, KY631492, KY648934, KY765317, KY765318, KY765320, KY765321, KY765322, KY765323, KY765324, KY765325, KY765326, KY765327, KY785410, KY785415, KY785418, KY785419, KY785420, KY785422, KY785423, KY785426, KY785427, KY785435, KY785441, KY785442, KY785445, KY785448, KY785450, KY785452, KY785453, KY785455, KY785459, KY785464, KY785465, KY785466, KY785468, KY785469, KY785472, KY785473, KY785475, KY785476, KY785479, KY785480, KY785484, MF438286, NC_012532.

## Author Contributions

GW designed this study. TS analyzed the data and wrote the manuscript. JP, SZ, and ZX contributed to data collection. All of the authors approved the final manuscript.

## Funding

This work was supported by grants from Epidemiology, Early Warning and Response Techniques of Major Infectious Diseases in the Belt and Road Initiative (#2018ZX10101002), the National Natural Science Foundation of China (#81871699), and the Foundation of Jilin Province Science and Technology Department (#172408GH010234983).

## Conflict of Interest

The authors declare that the research was conducted in the absence of any commercial or financial relationships that could be construed as a potential conflict of interest.

## Abbreviations

ZIKV, Zika virus; NS5, non-structural protein 5; MCC, maximum clade credibility.

## Supplementary Material

Table S1 List of the 108 complete NS5 sequences of ZIKV strains available from GenBank that were selected to generate the ZIKV MCC (XLSX 12kb).
